# Supplementary material for: Mitochondrial dynamics and mitophagy are necessary for proper invasive growth in rice blast
Source: Mol Plant Pathol. 2019 Jun 20;20(8):1147–62. doi: 10.1111/mpp.12822 (PMC6640187; doi:10.1111/mpp.12822)
Supplement: Supplementary file 1 — Fig. S1 Mitochondrial morphology during appressorium formation in M. oryzae. [file MPP-20-1147-s001.pdf]

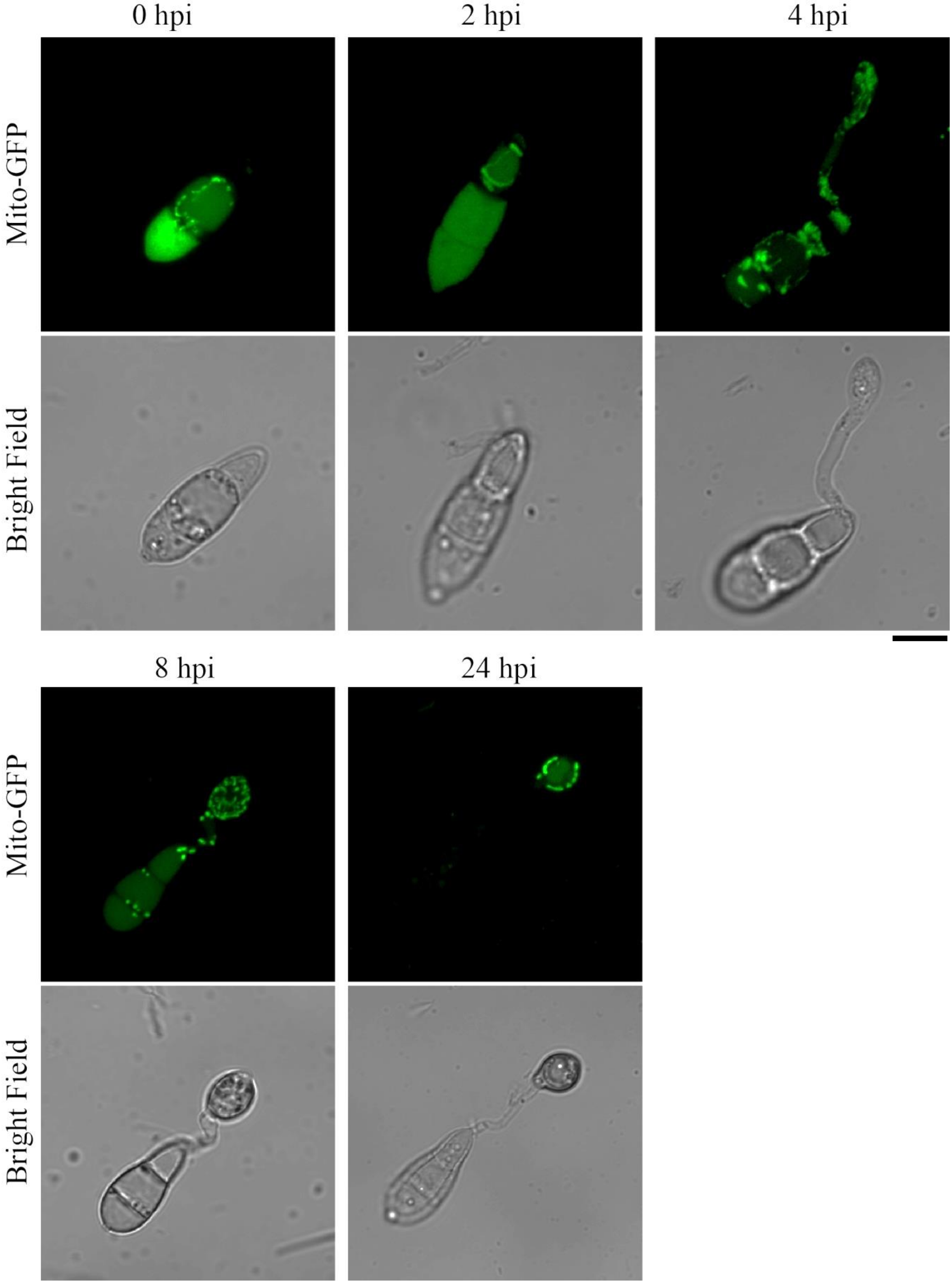

Fig. S1 Mitochondrial morphology during appressorium formation in *M. oryzae*. The conidial suspension of the *Mito-GFP* strain was inoculated on the inductive surface. Confocal microscopy was carried out at 0, 2, 4, 8, and 24 hpi. Scale bar: 10 μm
