# Supplementary figures and images for: Mitochondrial dynamics and mitophagy are necessary for proper invasive growth in rice blast
Source: Mol Plant Pathol. 2019 Jun 20;20(8):1147–62. doi: 10.1111/mpp.12822 (PMC6640187; doi:10.1111/mpp.12822)

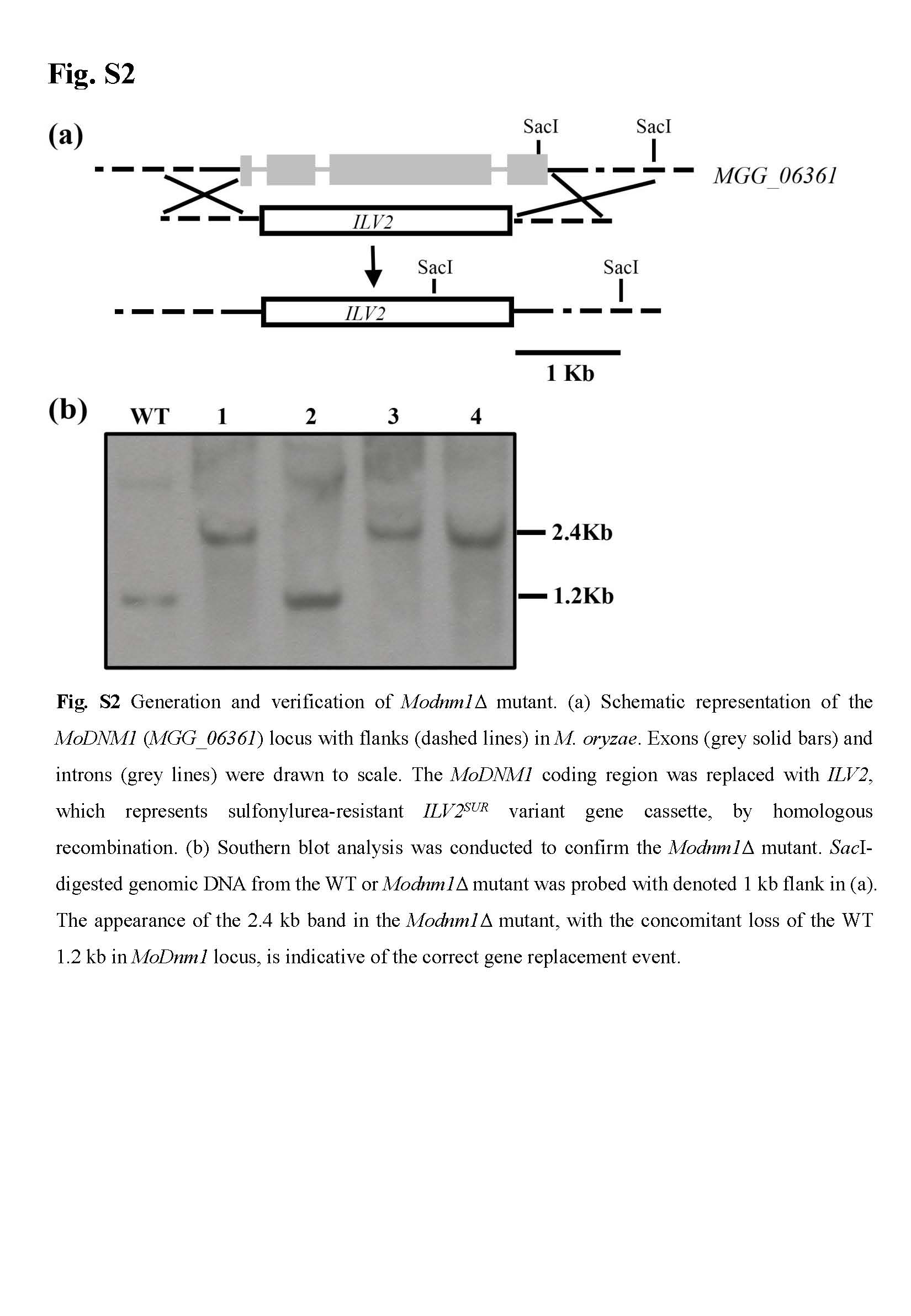

Supplement: Supplementary file 2 — Fig. S2 Generation and verification of Modnm1Δ mutant. [file MPP-20-1147-s002.jpg]

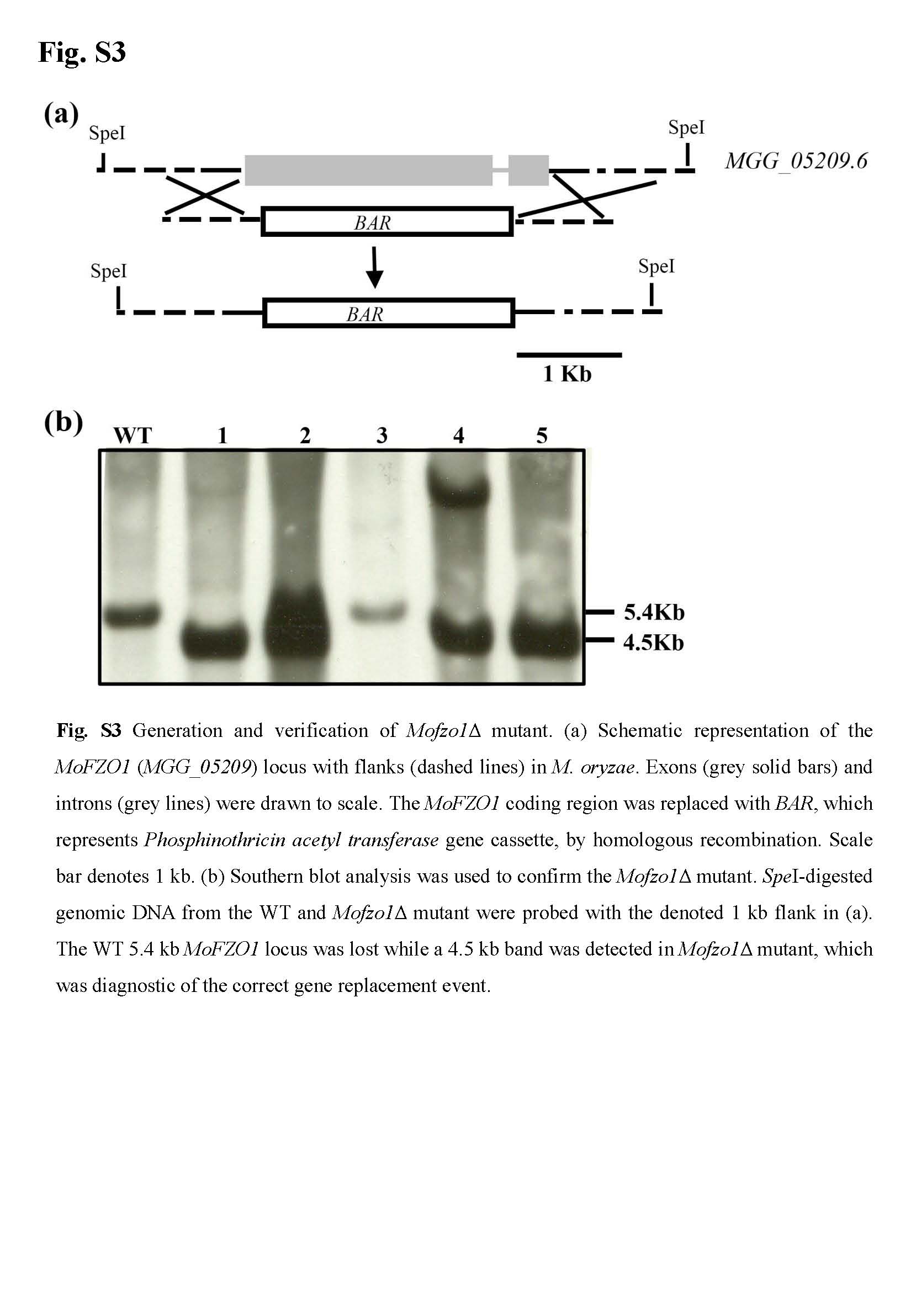

Supplement: Supplementary file 3 — Fig. S3 Generation and verification of Mofzo1Δ mutant. [file MPP-20-1147-s003.jpg]

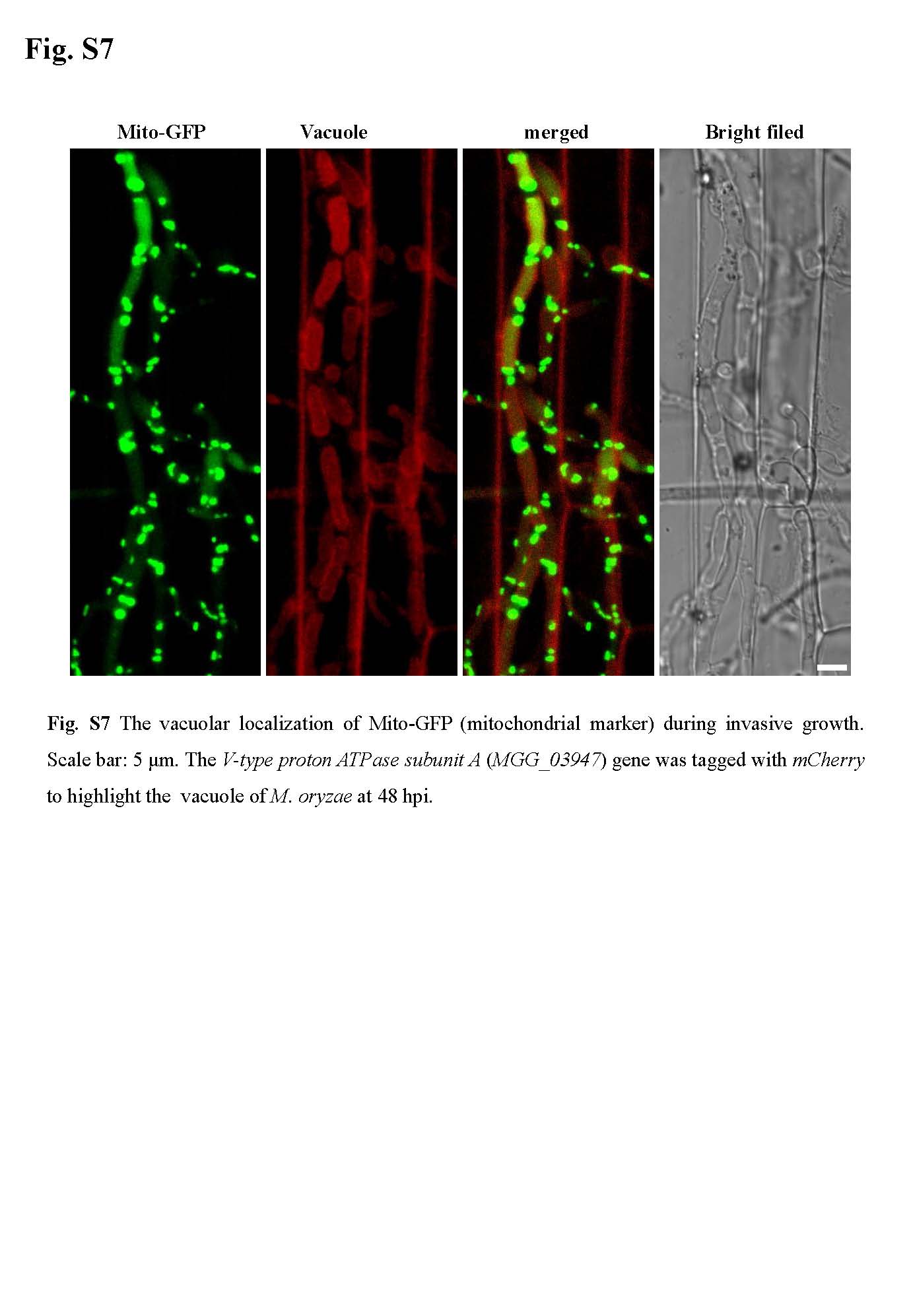

Supplement: Supplementary file 7 — Fig. S7 The vacuolar localization of Mito‐GFP (mitochondrial marker) during invasive growth. [file MPP-20-1147-s007.jpg]

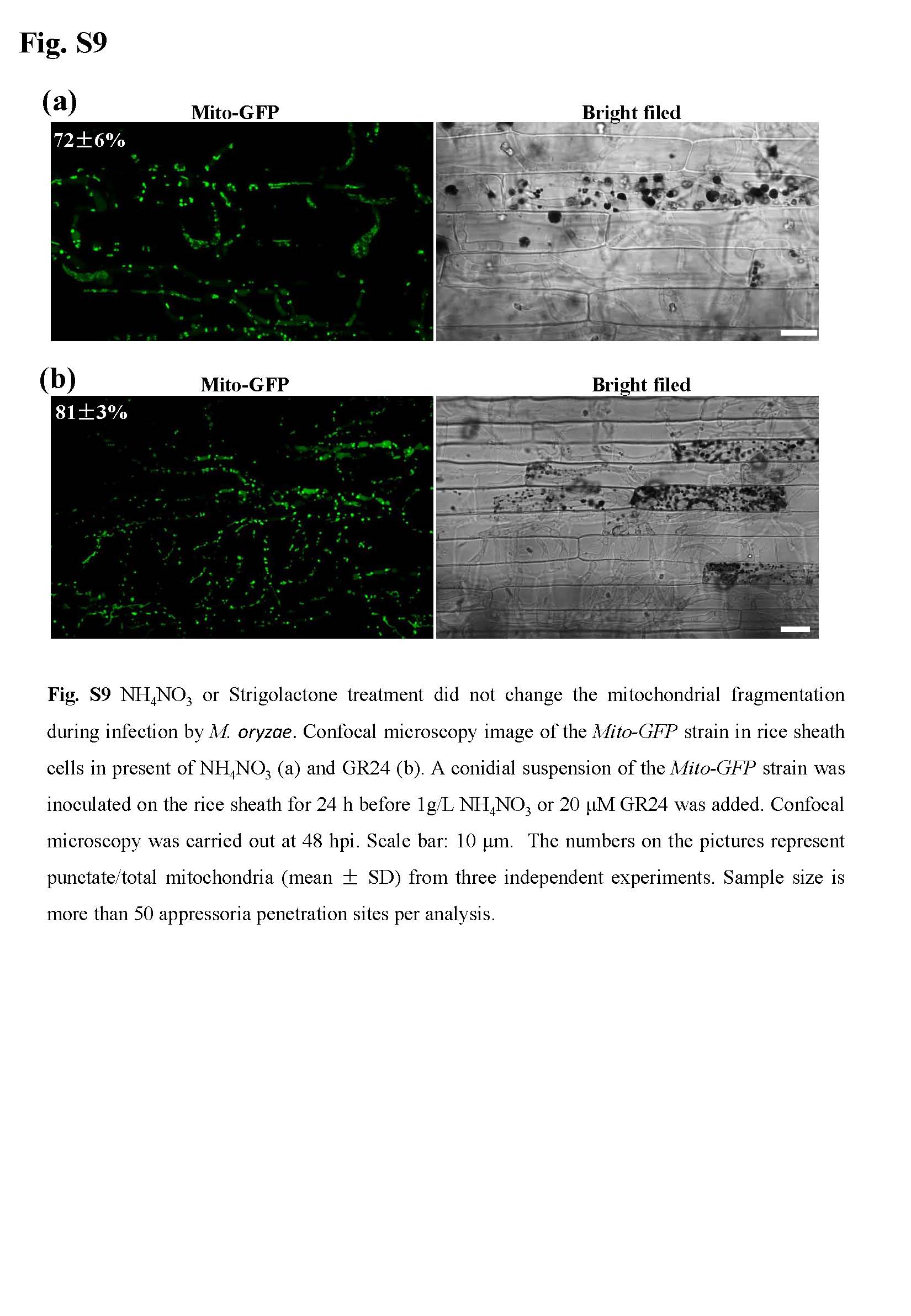

Supplement: Supplementary file 9 — Fig. S9 NH4NO3 or strigolactone treatment did not change the mitochondrial fragmentation during infection by M. oryzae. [file MPP-20-1147-s009.jpg]

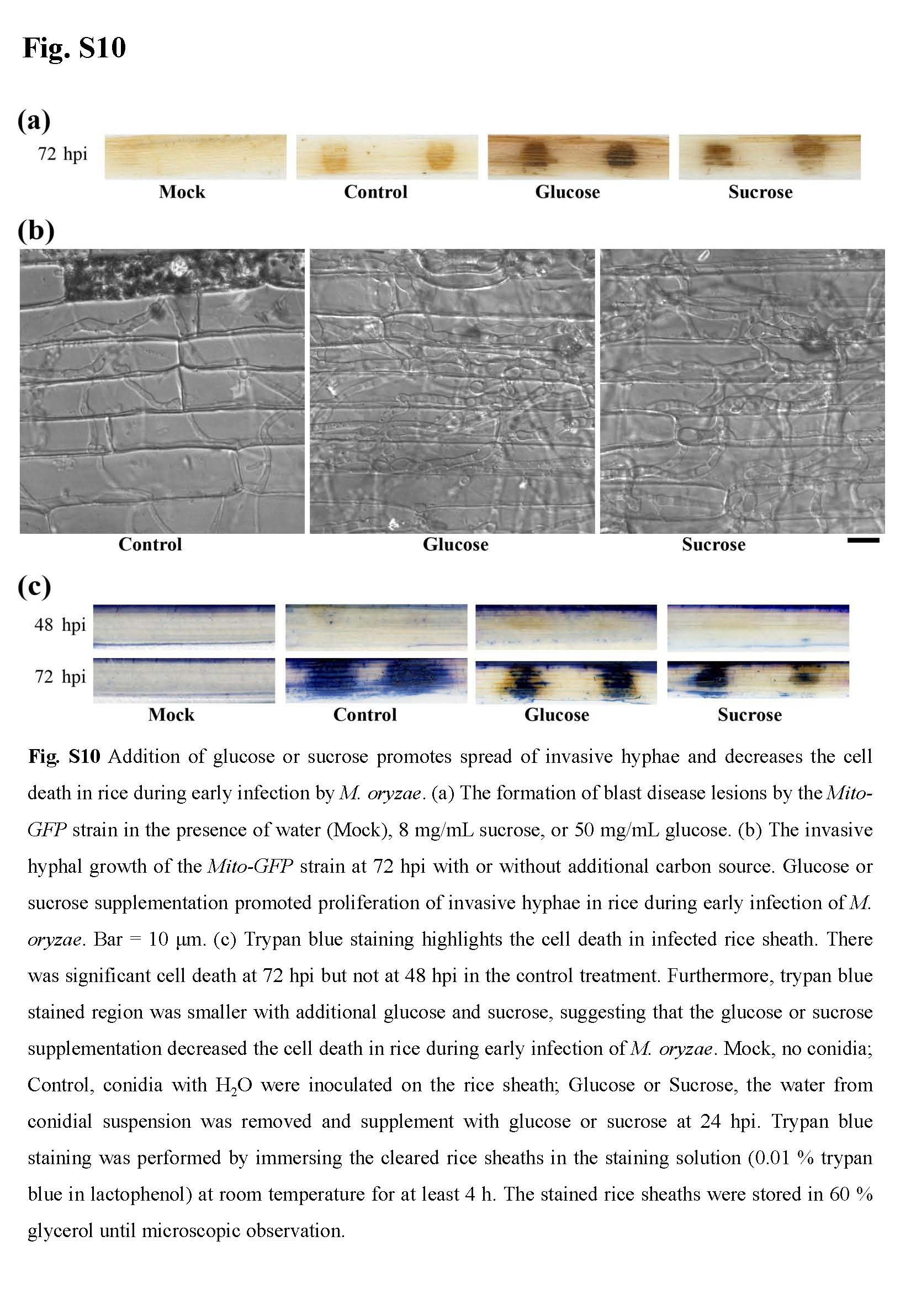

Supplement: Supplementary file 10 — Fig. S10 Addition of glucose or sucrose promotes spread of invasive hyphae and decreases the cell death in rice during early infection by M. oryzae. [file MPP-20-1147-s010.jpg]
