# Supplementary material for: Mitochondrial dynamics and mitophagy are necessary for proper invasive growth in rice blast
Source: Mol Plant Pathol. 2019 Jun 20;20(8):1147–62. doi: 10.1111/mpp.12822 (PMC6640187; doi:10.1111/mpp.12822)
Supplement: Supplementary file 4 — Fig. S4 Oxidant treatment induces mitochondrial fragmentation. [file MPP-20-1147-s004.pdf]

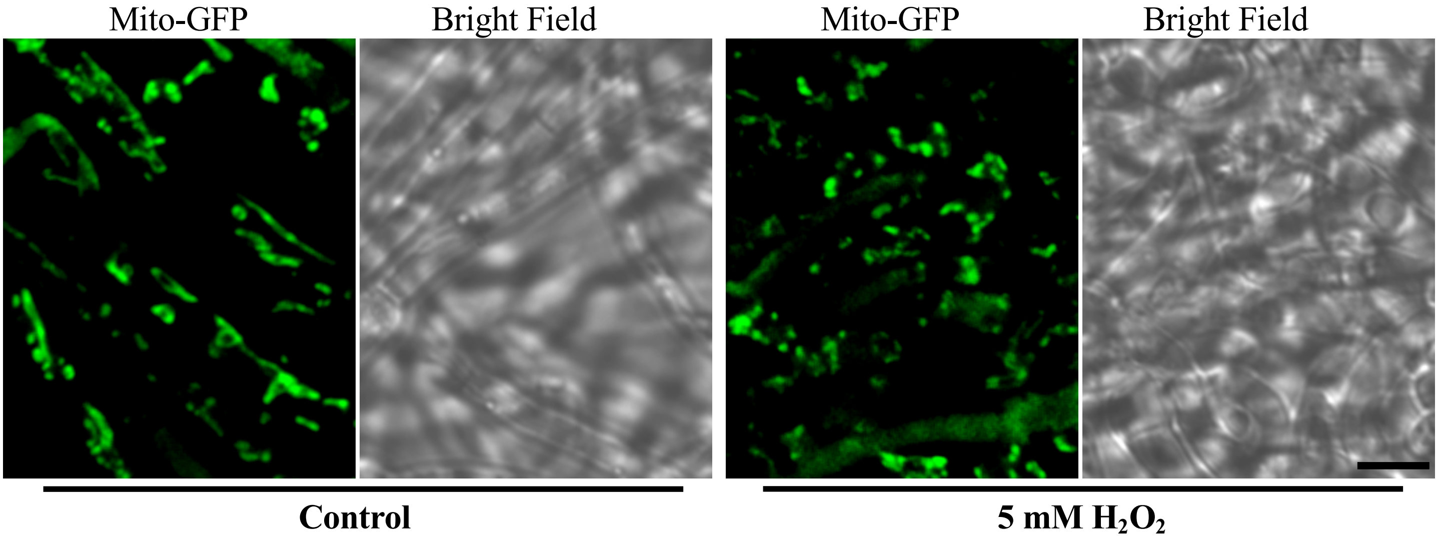

Fig. S4 Oxidant treatment induces mitochondrial fragmentation. The *Mito-GFP* strain was grown in liquid CM for 2 d followed by inoculation in for CM with 5 mM H_2O_2 for 12 h. Scale bar = 5 μm.
