# Supplementary material for: Mitochondrial dynamics and mitophagy are necessary for proper invasive growth in rice blast
Source: Mol Plant Pathol. 2019 Jun 20;20(8):1147–62. doi: 10.1111/mpp.12822 (PMC6640187; doi:10.1111/mpp.12822)
Supplement: Supplementary file 5 — Fig. S5 Antioxidant treatment does not delay mitochondrial dynamics during invasive growth. [file MPP-20-1147-s005.pdf]

Fig. S5

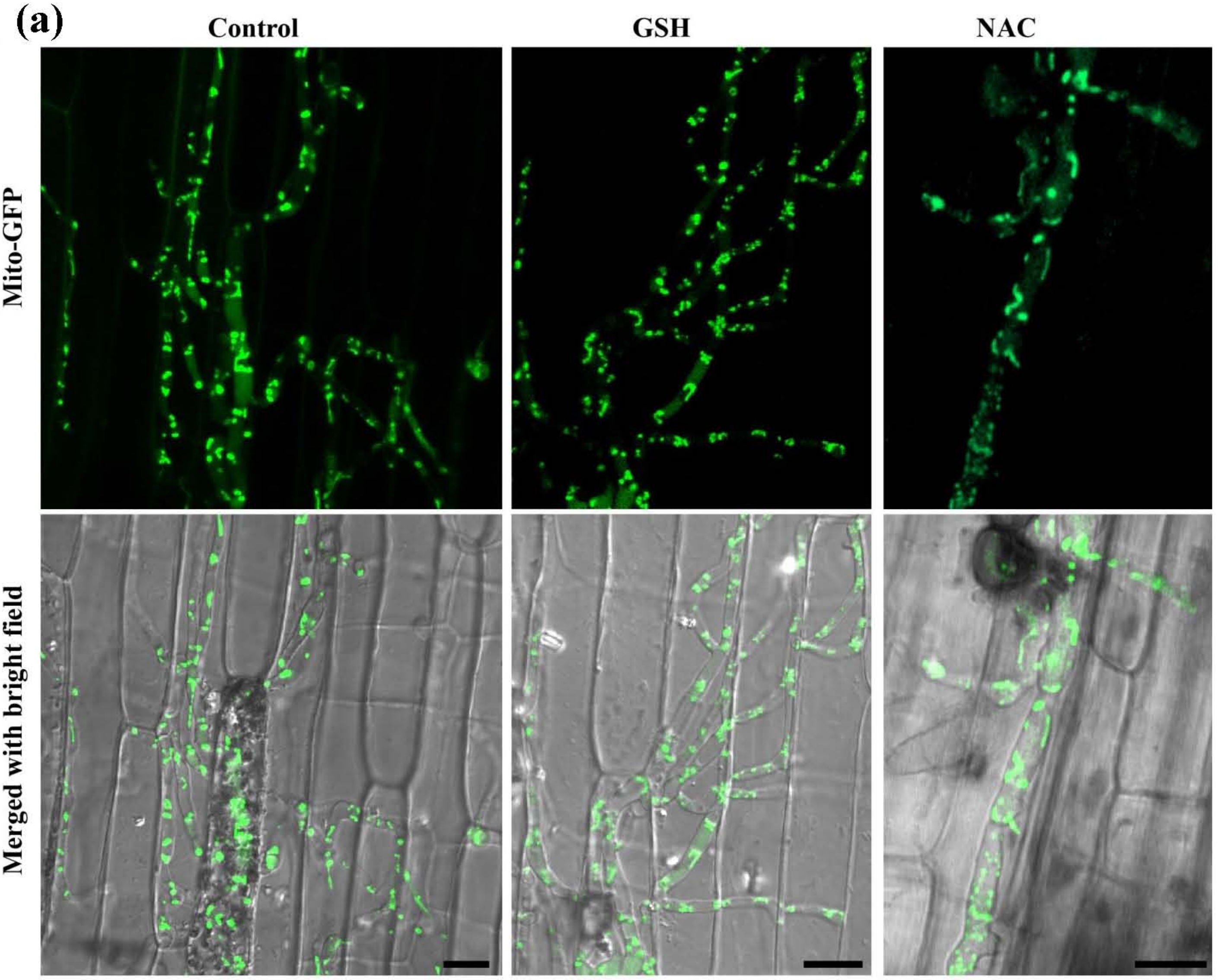

(a)
Control
GSH
NAC
Mito-GFP
Merged with bright field

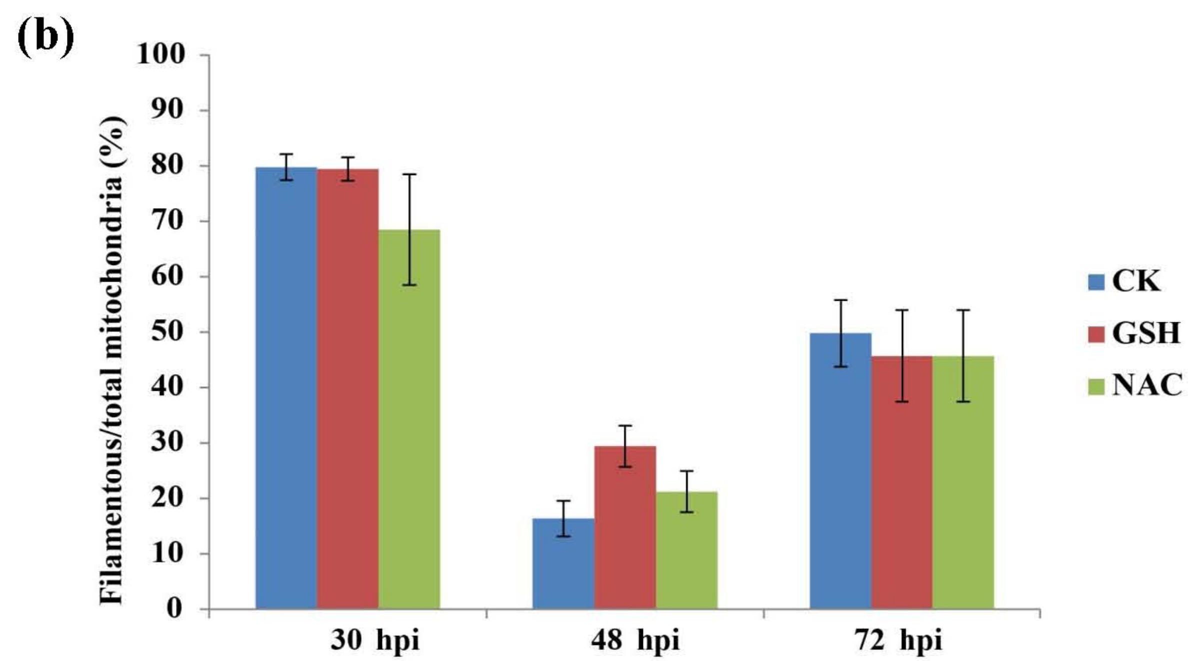

(b)
Filamentous/total mitochondria (%)
100
90
80
70
60
50
40
30
20
10
0
30 hpi
48 hpi
72 hpi
CK
GSH
NAC

Fig. S5 Antioxidant treatment does not delay mitochondrial dynamics during invasive growth. (a) Confocal microscopy image of the *Mito-GFP* strain in rice sheath cells in present of GSH or NAC. A conidial suspension of the *Mito-GFP* strain was inoculated on the rice sheath for 24 h before 2.5 mM GSH or 40 mM NAC was added. Confocal microscopy was carried out at 48 hpi. Scale bar: 10 μm. (b) Quantitative analysis of mitochondria of the *Mito-GFP* strain in rice sheath cells with GSH or NAC treatment. Values represent punctate/total mitochondria (mean ± SD) from three independent experiments. Sample size is more than 50 appressoria penetration sites per analysis.
