# Supplementary material for: Mitochondrial dynamics and mitophagy are necessary for proper invasive growth in rice blast
Source: Mol Plant Pathol. 2019 Jun 20;20(8):1147–62. doi: 10.1111/mpp.12822 (PMC6640187; doi:10.1111/mpp.12822)
Supplement: Supplementary file 6 — Fig. S6 Carbon source depletion induces mitochondrial fragmentation in M. oryzae. [file MPP-20-1147-s006.pdf]

Fig. S6

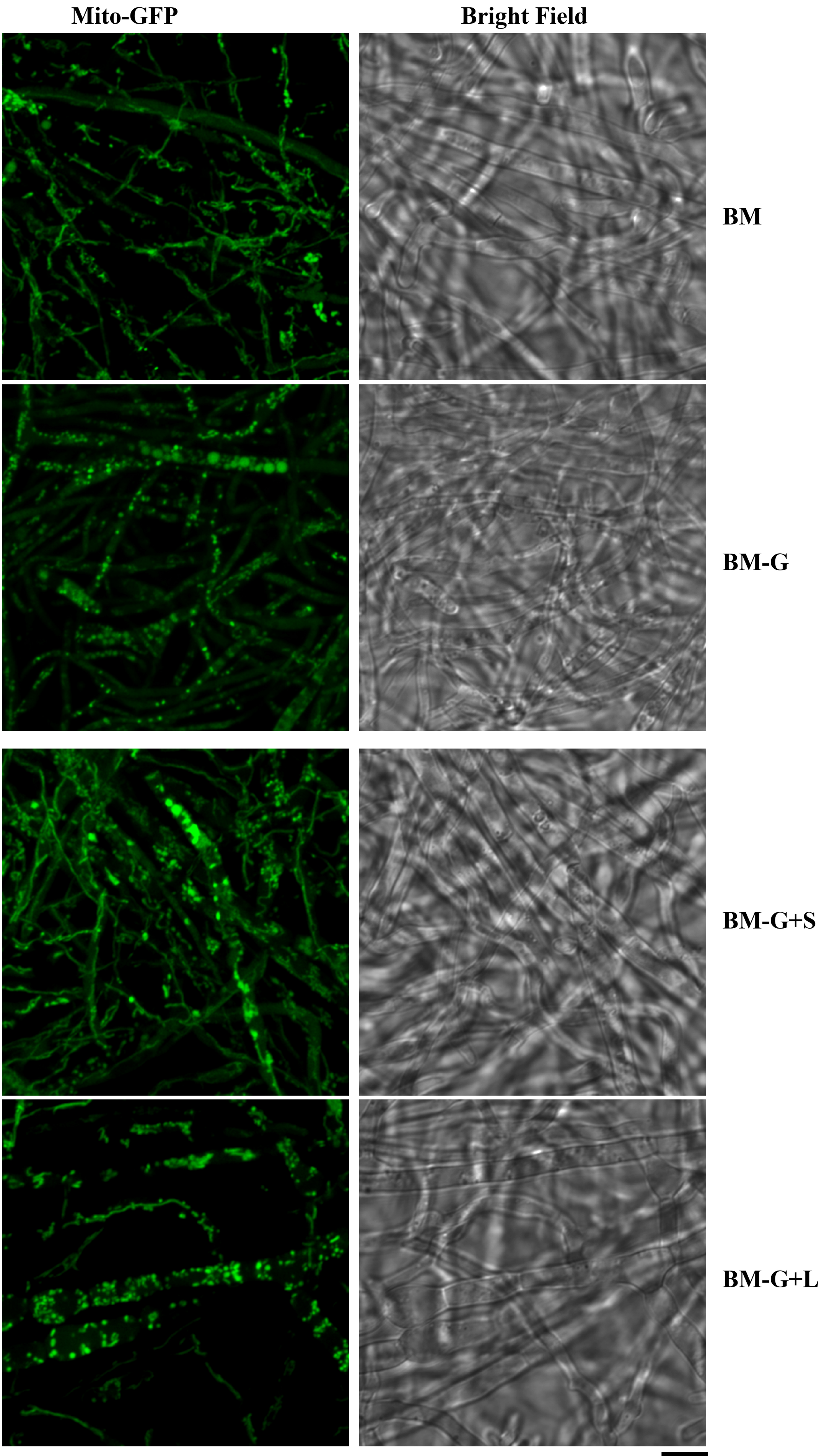

Fig. S6 Carbon source depletion induces mitochondrial fragmentation. Mitochondrial morphology upon carbon starvation. The results showed that carbon starvation could indeed induce the breakdown of mitochondrial network and led to increased accumulation of punctate mitochondria in *M. oryzae*. The *Mito-GFP* strain was grown in liquid CM for 1 d followed by inoculation in liquid BM (Basal medium; 1.6 g/L Yeast N2 base, 2 g/L L-Asparagine, 1 g/L NH_4NO_3, 10 g/L glucose, pH 6), BM-G (Basal medium lacking glucose), BM-G+S (Basal medium lacking glucose and supplemented with 10 g/L sucrose), and BM-G+L (Basal medium lacking glucose and supplemented with 10 g/L Lactose) for 2 d. Scale bar = 5 μm.
