# Supplementary material for: Mitochondrial dynamics and mitophagy are necessary for proper invasive growth in rice blast
Source: Mol Plant Pathol. 2019 Jun 20;20(8):1147–62. doi: 10.1111/mpp.12822 (PMC6640187; doi:10.1111/mpp.12822)
Supplement: Supplementary file 8 — Fig. S8 Prolonged nutrient starvation induces mitochondrial fragmentation and mitophagy. [file MPP-20-1147-s008.pdf]

Fig. S8

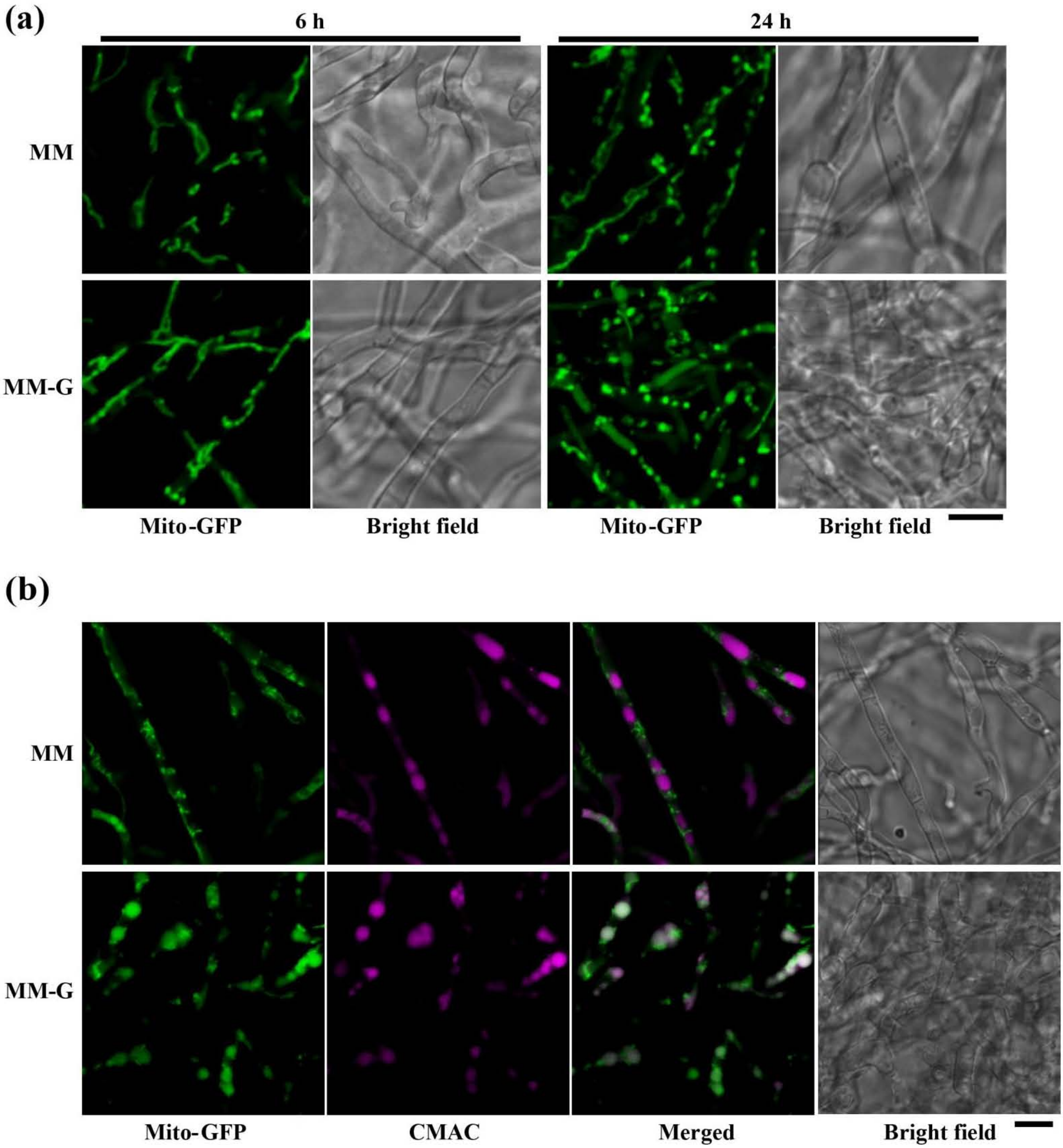
(a)
6 h
24 h
MM
MM-G
Mito-GFP
Bright field
Mito-GFP
Bright field
(b)
MM
MM-G
Mito-GFP
CMAC
Merged
Bright field

Fig. S8 Prolonged nutrient starvation induces mitochondrial fragmentation and mitophagy. (a) Mitochondrial morphology under carbon starvation. The *Mito-GFP* strain was grown in liquid CM for 2 days followed by inoculation in liquid MM (Minimal medium; 6 g/L $NaNO_3$, 0.5 g/L $MgSO_4$, 0.5 g/L KCl, 1.5 g/L KH_2PO_4, 10 g/L glucose, 0.1% (v/v) trace elements, pH 6.5) or MM-G (Minimal medium lacking glucose; 6 g/L $NaNO_3$, 0.5 g/L $MgSO_4$, 0.5 g/L KCl, 1.5 g/L KH_2PO_4, 0.1% (v/v) trace elements, pH 6.5) for 6 h or 24 h. The fragmented mitochondria are trafficked to the vacuoles for degradation upon carbon starvation for 24 h. (b) Mitophagy is induced by nutrient starvation for 24 h. The vacuoles in invasive hyphae were stained by CMAC and rendered in pseudo-color using Image J. Scale bar = 5 μm.
